# Multi-purpose, multi-level feature modeling of large-scale industrial software systems

**DOI:** 10.1007/s10270-016-0564-7

**Published:** 2016-10-14

**Authors:** Daniela Rabiser, Herbert Prähofer, Paul Grünbacher, Michael Petruzelka, Klaus Eder, Florian Angerer, Mario Kromoser, Andreas Grimmer

**Affiliations:** 1 Christian Doppler Laboratory MEVSS, Institute for Software Systems Engineering, Johannes Kepler University Linz, Linz, Austria; 2 Institute for System Software, Johannes Kepler University Linz, Linz, Austria; 3 KEBA AG, Linz, Austria

**Keywords:** Feature modeling, Large-scale software systems, Case study

## Abstract

Feature models are frequently used to capture the knowledge about configurable software systems and product lines. However, feature modeling of large-scale systems is challenging as models are needed for diverse purposes. For instance, feature models can be used to reflect the perspectives of product management, technical solution architecture, or product configuration. Furthermore, models are required at different levels of granularity. Although numerous approaches and tools are available, it remains hard to define the purpose, scope, and granularity of feature models. This paper first reports results and experiences of an exploratory case study on developing feature models for two large-scale industrial automation software systems. We report results on the characteristics and modularity of the feature models, including metrics about model dependencies. Based on the findings from the study, we developed FORCE, a modeling language, and tool environment that extends an existing feature modeling approach to support models for different purposes and at multiple levels, including mappings to the code base. We demonstrate the expressiveness and extensibility of our approach by applying it to the well-known Pick and Place Unit example and an injection molding subsystem of an industrial product line. We further show how our approach supports consistency between different feature models. Our results and experiences show that considering the purpose and level of features is useful for modeling large-scale systems and that modeling dependencies between feature models is essential for developing a system-wide perspective.

## Introduction and motivation

Feature modeling was originally proposed as part of the FODA method to elicit and represent commonalities and variability of systems’ capabilities in a specific domain [41]. Feature models define features—the end users’ (and customers’) understanding of the general capabilities of systems in a domain—and their relationships. Feature models, and variability models in more general, are nowadays widely used to capture the knowledge of domain experts regarding customer-facing features, system capabilities and properties, as well as configuration options [52, 58]. The term feature is commonly used by customers, product managers, and engineers to communicate about product capabilities and characteristics [8]. However, although numerous approaches and tools are available [18], defining the purpose, scope, and granularity of feature models remains hard, specifically when modeling large-scale industrial software systems.

Regarding the *purpose* of feature models, researchers have distinguished different modeling spaces [17, 42]: *problem space* features generally refer to systems’ specifications established during domain analysis and requirements engineering; *solution space* features refer to the concrete implementation of systems created during development, often by defining mappings of the features to code, whereas *configuration space* features exist to ease the derivation of products by resolving variability. Regarding the *scope* of feature models in large-scale systems, there is consensus that single monolithic feature models are inadequate to deal with the complexity of industrial systems [24, 44, 62]. This has led to the development of multi-product line approaches that support modularizing feature models in various ways [39]. Similarly, it has been shown that feature models vary with respect to their *granularity*, e.g., to distinguish high-level system features from lower-level capabilities. Moreover, *dependencies* between different feature models need to be managed [46]. For instance, it is often unclear how problem space features describing customer-facing capabilities and their variability are related to solution space features implementing this functionality; or how configuration space features are related to configuration options used by service engineers for customizing and fine-tuning a system.

In this paper, we (1) present an exploratory study on developing feature models for two large-scale software systems in the domain of industrial automation. The study, conducted as part of an ongoing research cooperation between the industrial and academic authors of this paper allowed us to investigate the purpose, scope, and granularity of feature models but also to elicit modeling language requirements addressing the characteristics and needs of large-scale industrial software systems. (2) We propose FORCE, a modeling approach addressing the requirements by supporting multi-purpose, multi-level feature modeling, including the definition of model dependencies and mappings of features to the code base. (3) Furthermore, we adapted and extended the FeatureIDE [70], an Eclipse-based feature modeling tool, to support our approach. Our tool architecture also integrates a program analysis framework we developed to support the IEC 61131-3 standard, a non-mainstream family of languages used in the industrial automation domain [33]. (4) Finally, we demonstrate the expressiveness and extensibility of our approach by applying it to the Pick and Place Unit (PPU) [14, 73], a manufacturing system described in the literature, and a subsystem of an industrial product line for injection molding machines. We also show how consistency can be ensured during modeling.

Only few reports are available on how to structure and organize different feature models, and what kind of dependencies need to be considered. In particular, there is a lack of guidelines on feature modeling in large-scale systems. Organizations moving toward a product line approach or feature-oriented development paradigm can benefit from examples and lessons learned when planning their own modeling approach. Our work can be useful for practitioners modularizing feature models and managing dependencies between features in the problem space, solution space and configuration space.

Our paper is based on an earlier conference publication [50] that described the experiences and lessons learned in our exploratory study. We extended this work in several ways: We describe the tool-supported FORCE modeling approach we developed based on the results of the case study, including details on feature-to-code mappings. We further present a multiple case study to validate our approach on two examples of product lines for industrial automation. Our results show that FORCE allows building features models including dependencies between features as well as feature-to-code mappings in different modeling spaces. We further demonstrate how such dependencies can be used to check FORCE models regarding consistency.

The paper is organized as follows: In Sect. 2, we briefly describe the industrial background and motivation. Section 3 motivates and describes our overall research approach. Section 4 presents the exploratory case study we conducted to investigate the industrial context and to derive requirements for our modeling approach. Section 5 presents the FORCE modeling approach. Section 6 describes the FORCE tool environment. Section 7 presents the application of our approach to two case study systems. Section 8 discusses experiences and lessons learned. Section 9 relates our work with existing research on variability modeling of large-scale systems. Section 10 rounds out the paper with a conclusion and an outlook on future work.

## Features in industrial automation systems: background and motivation

Our industry partner Keba develops and produces industrial automation solutions for customers worldwide (http://www.keba.com). The company’s product portfolio includes control solutions for injection molding machines, robotics, and heating systems. Their products exist in numerous variants to address requirements of different customers and market segments. Keba is currently exploring the benefits of the feature-oriented software development paradigm, which is seen as promising to ease software maintenance, to create awareness for feature reuse, to automate product derivation, and to improve documentation.

As part of a research cooperation, we recently studied the development practices of Keba’s KeMotion and KePlast product lines [8, 48–50].^1^ The data collected in workshops and interviews with Keba ’s senior developers, software architects, and product managers allow us to better understand the industrial context for feature modeling in the large.

### Different views on features

The term feature is widely used in the company to communicate during development and maintenance, independent of the specific methods and technologies used. Obviously, the meaning of the term depends on role-specific perspectives and needs in Keba ’s current development process: For instance, *sales people* identify the needs of potential customers in terms of new system features. *Product managers* drive the development of different KeMotion and KePlast product variants by defining product line features addressing market needs. They use features to define the scope of products from a market and customer perspective. They document problem space features in product maps, i.e., matrices that allow comparing related products over numerous features. These spreadsheets comprise high-level system features, feature associations, available hardware options, and references to order numbers used by sales people. At the more technical level, *software engineers* work with solution space features, i.e., pieces of the code implementing a specific functionality denoted by a feature. Features, however, are often crosscutting and can span multiple components, sub-systems, and languages. Engineers use a wide range of mechanisms to implement feature variability, e.g., interfaces to hook in new functionality; capabilities for adding, exchanging, or reloading modules; or support for connecting to specific hardware equipment. *Architects* use UML class diagrams for modeling and documenting solution space features. Finally, Keba uses a custom-developed configurator that defines configuration space features guiding the derivation of products from their product lines [51]. Their tool allows deriving initial products, which are then customized and adapted by developers, e.g., by adding new features to meet the customers requirements. Finally, *commissioning engineers* fine-tune systems by calibrating the properties of features.

### Different forms of features

Features also exist in different forms, covering a wide range of notations and tools. We use the KePlast feature MoldCavityPressureSensor for illustration. In injection molding machines, the polymer raw material is injected into a mold to shape it into the desired form [53]. Molds can have single or multiple cavities. In multiple cavity molds, cavities can be identical and form equal parts; however, cavities can also be unique and form multiple different parts [63]. Cavity sensing is used to provide a quality index of the injection-molded part. A pressure signal is used for determining whether the cavity pressure curve is repeatable between shots. The measured cavity pressures indicate the quality of the produced parts. In case of anomalies, it is likely that the quality of the produced parts degrades.

**Fig. 1:**
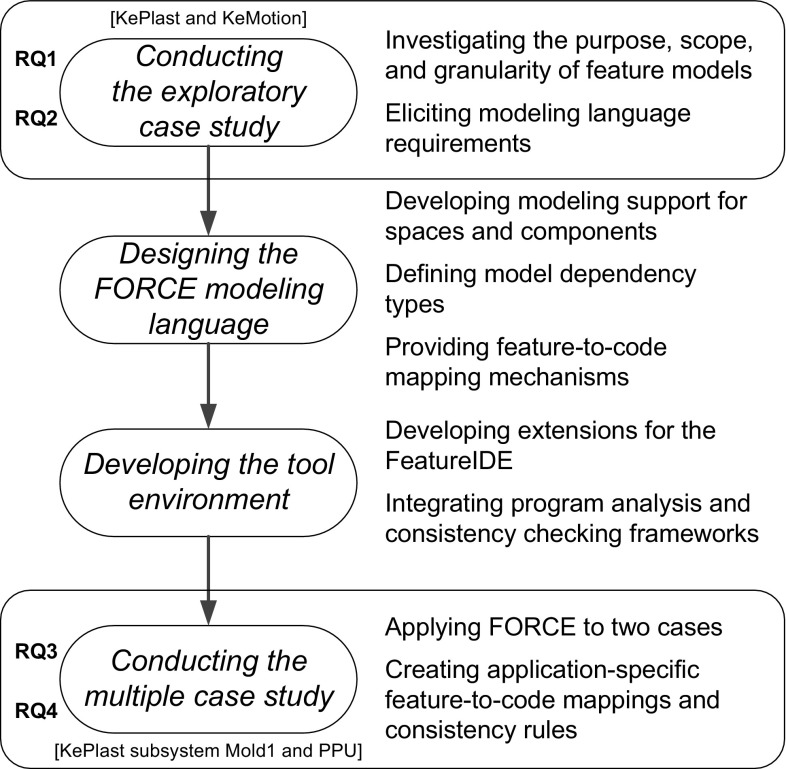
Research approach

Representations of the feature MoldCavityPressureSensor can be found in each modeling space and traces exist in spreadsheets, KePlast platform code, and the source code of the configuration tool. The feature is documented in the problem space as an option in the KePlast product map. Specific code exists in the solution space for implementing the feature. First, there is a variable hw_CavityPressure, which serves as the endpoint to the hardware sensor. The variable declaration is optional and only included if the feature is enabled to activate additional code for handling cavity pressure measurement. Further, if enabled, the sensor is also shown on several user interface masks. Finally, regarding the configuration space, the custom-developed configurator allows selecting up to four sensors for measuring cavity pressure values. The example further shows that although different stakeholder roles manage the feature well for different purposes, only few links exist between system features in spreadsheets, options in configuration tools, and features as implemented in source code.

## Research approach

Our research approach covers four phases, which are depicted in Fig. 1 together with the research questions and study subjects. Specifically, following the categories presented by Easterbrook et al. [26], we first conducted an exploratory case study, which allowed us to investigate phenomena on features in our domain of interest. Based on our findings, we developed a modeling approach, which we tested in a second confirmatory case study. We describe the detailed case study processes and results in Sects. 4 and 7, following existing schemes of conducting and reporting case study research [64, 74].

**Phase 1—Exploratory case study.** The *objective* of the first case study was to investigate to what extent multi-purpose, multi-level feature models are useful in industrial systems. The study, conducted as part of an ongoing research cooperation between the industrial and academic authors of this paper, allowed us to investigate the purpose, scope, and granularity of feature models and to elicit modeling language requirements addressing the characteristics of large-scale industrial software systems. Following Runeson and Höst [64] and Easterbrook et al. [26], we classify the research in this phase as partly exploratory and partly descriptive, as we focus on finding out to what extent multi-purpose, multi-level feature models are useful. We also portray the situation by reporting characteristics of specific modeling spaces.

The selected *cases* are Keba’s industrial automation systems KeMotion and KePlast. KeMotion (2.7 million LoC) is a control system for robotics, comprising a software platform as well as hardware control units and mobile display units. KeMotion covers the entire motion spectrum of the robot, covering track-consistent, shortest possible point-to-point movements or driving of individual robot axes. The system offers all types of interpolation, unlimited in the 6D space (position and orientation). Besides its motion capabilities, the system also offers guided programming and execution of robot sequences. KePlast (3.8 million LoC) is a comprehensive platform for the automation of injection molding machines, comprising a configurable control software framework, a visualization system, programming tools, and a configuration tool to customize solutions based on existing components and variants. The platform exists in several variants, e.g., there is one specific variant for the Chinese market.

Specifically, we pursued two *research questions*:

*RQ1—How useful are multi-purpose, multi-level feature models for large-scale industrial systems?* We explored whether feature models for large-scale, real-world systems can be organized in terms of distinct modeling spaces and multiple modeling levels.

*RQ2—What are the characteristics of specific modeling spaces?* We modeled specific areas of the systems in detail to gain in-depth results of selected feature models.

**Phase 2—Definition of the** FORCE **modeling language.** Based on the modeling language requirements, we designed the modeling language FORCE to support multi-purpose, multi-level feature modeling, including dependencies between feature models and mechanisms allowing to map features to the code base.

**Phase 3—Tool development.** We developed the FORCE modeling environment to support our approach. In particular, we adapted and extended the FeatureIDE [70], an Eclipse-based feature modeling tool. Our tool architecture also integrates a program analysis framework we developed to support the IEC 61131-3 standard, a non-mainstream family of languages used in the industrial automation domain [33]. It further exploits a consistency checking framework [61, 71] for determining the consistency of FORCE models.

**Phase 4—Multiple case study.** The *objective* of the second case study was to demonstrate the expressiveness and extensibility of the FORCE modeling approach by applying it to different contexts. Following Easterbrook et al. [26], we classify this phase of research as *confirmatory*, as our goal was to test our approach in a realistic context.

We applied FORCE to two *cases*: the PPU system [14, 73] and KePlast ’s subsystem Mold1. The PPU system is a well-known example of a manufacturing system for material handling and sorting of different workpieces. It is described by Vogel-Heuser et al. [73] as an open case study for studying the evolution of automation systems, which exist in various configurations. The Mold1 subsystem operates an injection molding machine’s mold when producing plastic parts. It comprises 15,906 lines of IEC 61131-3 code and controls opening and closing the mold according to velocity profiles and target positions. It further prevents mold damage due to jammed plastic parts and hinders incorrect mold movement. Optionally, the component supports multiple cavity pressure sensors for building up and controlling mold pressure.

Specifically, we investigated two research questions:

*RQ3—How suitable is the* FORCE *approach to support multi-purpose, multi-level feature modeling?* Emphasizing depth over breadth, we developed feature models for the different modeling spaces of the PPU system and the software component Mold1 to validate the expressiveness of our approach.

*RQ4—How do the extensibility mechanisms of* FORCE *support application-specific feature-to-code mappings and consistency checking of models?* We developed extensions to support the variability implementation techniques used in the PPU system and in KePlast. We further discuss examples of using the dependencies in our FORCE tool for consistency checking rules to incrementally determine the validity of the models.

## Exploratory case study: feature modeling of KeMotion and KePlast

The goal of developing feature models for KeMotion and KePlast in case study was to explore to what extent multi-purpose, multi-level feature models are useful in industrial systems. We did not intend or aim to completely model the two systems. Specifically, regarding *RQ1—How useful are multi-purpose, multi-level feature models for large-scale industrial systems?—* we explored to what extent organizing feature models in terms of distinct modeling spaces and multiple modeling levels is feasible and sensible. This research question addresses specifically the breadth of the resulting models and the coverage of different spaces and levels. In particular, we report model metrics and insights related to modeling spaces, levels, and dependencies. Regarding *RQ2—What are the characteristics of specific modeling spaces?—* we modeled specific areas of the systems in detail to gain in-depth results of selected feature models. In particular, we describe detailed metrics measuring KePlast ’s problem space and configuration space models.

### Modeling process

Before we started the modeling phase, we conducted several preparatory steps:

*Analyzing representations of selected features in different modeling spaces.* We took a look at exemplary features of KeMotion and KePlast to better understand how these features are used for product management, product configuration, and during development, e.g., regarding different variability mechanisms. For instance, we investigated the KePlast feature ImpulseCounter needed for direct clamping in injection molding machines in all modeling spaces: In product management, the ImpulseCounter is represented as the standard function *automatic mold height adjust for direct clamping machines*. The feature can further be found in the product configuration tool, i.e., the common setup of the closure unit of an injection molding machine. At code level, the feature is reflected by a variable representing an endpoint to optional machine equipment and code handling the sensor measurement from the impulse counter. Studying selected features increased our confidence in the existence of the different modeling spaces.

*Prototypical modeling of selected subsystems.* We then created initial feature models for KeMotion following the concepts of the Common Variability Language (CVL) [16]. Specifically, we created five models comprising KeMotion ’s configuration space features and six models reflecting solution space features. We used SINTEF’s CVL 2 tool prototype [69] for this purpose as it adheres to the proposed CVL standard [16] and supports multiple interrelated feature models and configurable units. Although this tool was not mature enough for our purpose, the experience helped us to implement prototype extensions to the FeatureIDE tool suite [70], which we used in our modeling activities. Specifically, these extensions allowed us to manage multiple feature models representing physical components and logical components at different levels, as well as dependencies between interspace features.

The actual modeling process was performed in two steps:

*Modeling strategy and data sources.* Based on the CVL prototype models, we started modeling KeMotion and KePlast, following a top-down modeling strategy for both systems. For KeMotion, the problem space models were created as a first step, despite no detailed product map was available at that time (this was only started recently by the company). We then focused on modeling the configuration space and analyzed the configurator included in Keba ’s engineering tool suite. Finally, we explored the code base to find solution space features. The KeMotion system uses various variability mechanisms for optional and alternative features and special emphasis was put on investigating how those features are implemented. The author in charge of modeling the solution space has detailed knowledge of KeMotion ’s code base. The resulting models thus provide a good coverage of the code base; however, we did not complete them for all subsystems (cf. RQ1), and we also defined no mappings from the features to their implementation in the code base. For KePlast, we started with creating the problem space models based on an existing product map maintained by product managers. For creating the configuration space models, we investigated KePlast ’s custom-developed configuration tool. Finally, we created solution space models by exploring the code base of KePlast, again emphasizing breadth over depth. We investigated KePlast ’s variability mechanisms and found that features can be mapped to modules, classes, functions, or configuration options. For instance, features can be linked to program variables, which represent an initial seed activating a feature implementation. We investigated the different types of feature implementation mechanisms but did not model mappings of features to code, although the code was often inspected to understand the meaning of certain features. Moreover, we did not consider feature attributes that would be important for product derivation and feature selection.

*Model validation and analysis.* The author who created the multi-level feature models was involved with the KeMotion application for more than eight years and recently moved as a developer to the KePlast team. A second author cross-checked the created models and resulting metrics. The feature models were iteratively refined and validated in multiple discussions. Further, the created models were presented in a workshop with KeMotion and KePlast architects to get feedback and to clarify open issues. When cross-checking the feature models, we used a dictionary standardizing domain terminology [46]. For instance, the domain dictionary for injection molding describes the problem space features related to HotRunner as follows: *A hotrunner is used to maintain a molten flow of plastic from the injection molding machine nozzle to the gate in a plastic injection mold*. Such definitions were helpful to understand the meaning of features.

**Fig. 2:**
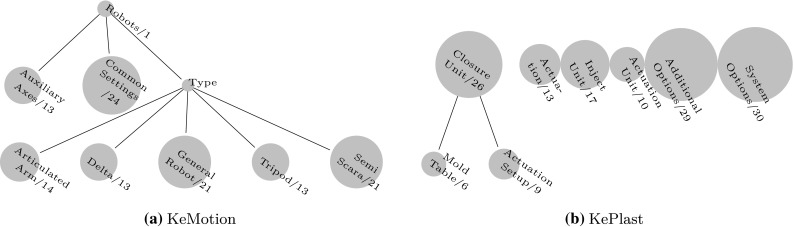
Configuration space feature models. During product configuration, configuration decisions are first taken for features in higher-level models, while lower-level models address more detailed configuration options. **a** KeMotion. **b** KePlast

We now present results on the usefulness of feature modeling with multiple modeling spaces and levels. We report system-wide model characteristics as well as detailed model characteristics for KePlast ’s problem and configuration space.

### RQ1 results

Regarding *RQ1—How useful are multi-purpose, multi-level feature models for large-scale industrial systems?*—we report metrics on feature model properties as proposed by Berger et al. [7]. More specifically, we measure the created variability models with structural metrics concerning the size and shape of the models. Table 1 summarizes the number of features per type (mandatory, optional, alternative, and modeling space), collections, and components, as well as interspace and intra-space dependencies.

**Table 1:** KeMotion and KePlast model characteristics

Characteristic	KeMotion	KePlast
Features	395	454
Mandatory	181	77
Optional	154	212
Alternative	60	165
Configuration space	120	140
Problem space	138	199
Solution space	137	115
Collections	48	52
Components	5	5
Interspace dependencies	29	40
Intra-space dependencies	5	38

We further provide bubble tree diagrams visualizing the size of feature models for the different modeling spaces and modeling levels for KeMotion and KePlast. For instance, Fig. 2a representing KeMotion ’s configuration space comprises the high-level model Robots (one feature), the second-level models AuxiliaryAxes (13 features) and CommonSettings (24 features), and third-level models covering configuration options for different robot types.

**Fig. 3:**
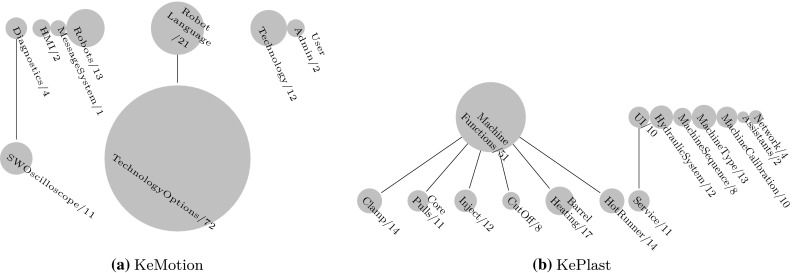
Problem space feature models. Top-level models define a high-level system capabilities, while lower-level models address detailed system characteristics. **a** KeMotion. **b** KePlast

**Fig. 4:**
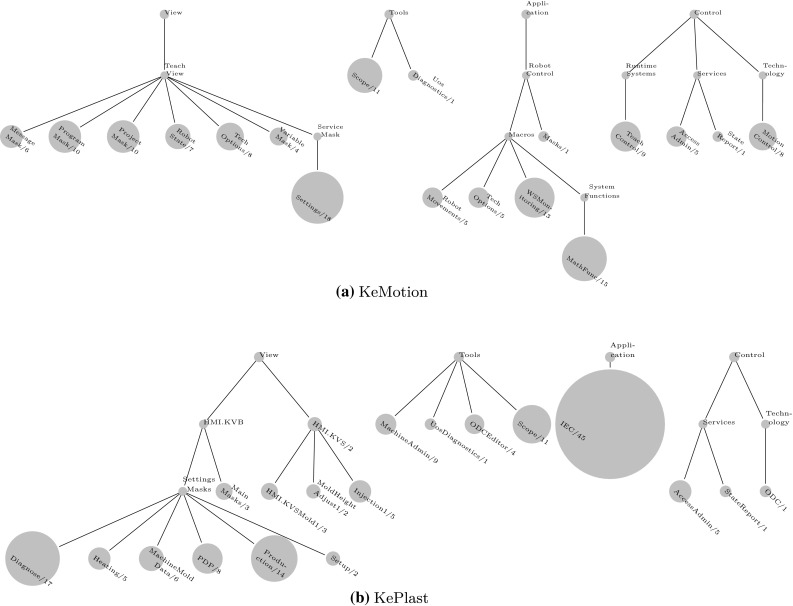
Solution space feature models. Top-level elements are higher-level system functions and collections for organizing the model. Low-level elements are fine-grained features and configuration settings. **a** KeMotion. **b** KePlast

*Modeling Spaces.* The results show that for both KeMotion and KePlast, features were modeled in all three modeling spaces. The configuration space models (cf. Fig. 2) define configuration decisions in the KeMotion and KePlast configurators, reflecting Keba ’s staged configuration process [19]. For instance, KeMotion ’s configuration space models for different robot types (e.g., Tripod, or SemiScara) eliminate configuration choices provided by GeneralRobot. KePlast ’s configuration space contains 9 % mandatory features while KeMotion ’s configuration space contains 33 % mandatory features. The higher number of mandatory features is caused by a number of core features reflecting characteristics of diverse robot types.

Figure 3 shows that some of the problem space models are quite large, reflecting the rich capabilities and operations of KeMotion ’s domain-specific language for programming robots (cf. TechnologyOptions and RobotLanguage) and KePlast ’s MachineFunctions. KePlast ’s top-level problem space models—e.g., MachineFunctions, MachineType, or HydraulicSystem—have been defined based on the KePlast product map. KePlast ’s problem space contains 22 % mandatory features representing standard functionality. Optional features typically require an extra license. KeMotion ’s problem space mainly covers commands of KeMotion ’s domain-specific language for programming robots. It contains 38 % mandatory features reflecting standardized commands.

The solution space models of both KeMotion and KePlast include several smaller feature models with less than 30 features, thus reflecting the modular design of the applications (cf. Fig. 4). KeMotion ’s solution space contains 36 % optional features defining capabilities of the robot programming language. Specific robot commands used in end user programs are activated only during load time; thus, the instruction set was modeled as optional features whose variability is bound at load time. KePlast ’s solution space on the other hand contains 82 % optional features. The inclusion of optional visualization system features often depends on configuration space features. For instance, the feature CalibCavPrSens13 reflecting the user interface for visualizing up to three cavity pressure sensors is included depending on the configuration space feature MoldCavityPressureSensor.

*Modeling levels.* Both models comprise around 50 components and collections (representing logical components), which establish a hierarchy of feature models. Collections and components were used frequently for structuring the models (with a nesting level ranging between two and five). However, the solution space models are an initial attempt to create feature-based abstractions of the source code, and further refactoring of the larger models will likely increase their depth. For instance, larger collections like TechnologyOptions, RobotLanguage, or MachineFunctions will possibly be re-modularized by extracting feature collections in separate feature models.

*Modeling dependencies.* Although revealing interspace dependencies was not our primary goal when creating the models, our experiences still show a lack of explicit knowledge about feature dependencies. The author creating the models added commonly known constraints. For instance, crosstree constraints in KePlast ’s configuration space were defined after analyzing KePlast ’s custom-developed configurator. The KeMotion and KePlast models comprise 69 interspace dependencies of different types, a first attempt for documenting relations between features in different spaces (see Sect. 5): 7 dependencies link problem space and configuration space, 33 link problem space and solution space, and 29 link configuration space and solution space.

### RQ2 results

Regarding *RQ2—What are the characteristics of specific modeling spaces?—* we report detailed model space characteristics about KePlast ’s problem and configuration space feature models, which are based on product maps and the custom-developed configurator, i.e., artifacts of high maturity.

**Table 2:** Model characteristics for KePlast configuration space  (CS) and problem space  (PS) models

Characteristic	KePlast CS	KePlast PS
Features	140	199
Mandatory	12	44
Optional	58	60
Alternative	70	95
Avg features per collection	17.5	13.3
Maximum depth of leaf features	6	5
Interspace dependencies	6	34
Intra-space dependencies	36	1
Features with cardinality	13	–

Table 2 summarizes the results related to RQ2. The maximum depth of leaf features considers both the depth of a feature model and the level of the modeling space, i.e., depth increases with the number of hierarchically nested collections above a specific feature model. The maximum depth is 6 for the configuration space and 5 for the problem space. Examples for configuration space feature models with a maximum depth are Actuation, ClosureUnit, ActuationSetup, and SystemOptions. Configuring KePlast requires high domain expertise. For instance, features modeled in the SystemOptions feature model (cf. Fig. 2b) are often related to the specific hardware equipment of an injection molding machine.

The six exemplary interspace dependencies modeled for KePlast ’s configuration space link configuration space features (e.g., FastCloseValve) with solution space features (e.g., Mold1FastClose). The intra-space dependencies (i.e., crosstree constraints) are also available in the custom-developed configurator; however, it could only be revealed by inspecting the tool’s source code. Most of these constraints are related to an injection molding machine’s actuation type (e.g., electrical or hydraulic). The problem space models comprise a more complete set of interspace dependencies, of which 18 are related to the feature model MachineFunctions, 4 are related to MachineSequence, 8 are related to MachineType, and 4 are related to UI.

Features with cardinality are especially relevant in KePlast ’s configuration space models. An example for a feature with cardinality is the MoldCavityPressureSensor, allowing up to 4 sensors measuring cavity pressures.

### Summary

Our findings show the need for a feature modeling approach capable of managing multiple modeling spaces and interspace dependencies between features. The approach also needs to support modularization to facilitate a divide-and-conquer modeling strategy, which is required to deal with the complexity of large-scale industrial systems. Further, solution space features have a direct correspondence to code, which should be made explicit to ease program understanding, maintenance and evolution.

Specifically, a modeling approach needs to support *feature models for different purposes* (requirement 1), allowing a modeler to distinguish customer-facing features, software capabilities, and configuration decisions. The modeling spaces proposed in the literature (e.g., [17, 42]) are useful to distinguish different types of feature models in complex systems. The results show that *feature models* are needed at *different levels* of abstraction and granuarity (requirement 2), to address the multilayered architecture of large-scale systems. For instance, product managers may need to describe groups of product features at different levels of granularity. This confirms earlier work, e.g., on hierarchical product lines [62], which also suggests the use of hierarchically organized variability models. Furthermore, the need for complex product configuration in multiple stages [19] calls for multiple levels of models in the configuration space. Modelers need to explicitly define *dependencies between feature models* of different purpose that exist at different levels (requirement 3). This confirms the need for existing approaches for modeling dependencies between different modeling spaces [23, 37], between models of one space [31, 38] or between different levels of abstraction [62, 68]. Finally, our experiences confirm the need for *mapping features to code* (requirement 4). Features in a feature diagram are just a label, and engineers want to know how the features manifest themselves in the underlying architecture and code base. Solution space features should thus be directly mapped to implementation elements to understand how and at what granularity features are implemented. Moreover, optional and alternative features should be associated with corresponding variability implementation mechanisms to show how options are implemented.

## The FORCE modeling approach

We designed the modeling language FORCE (**F**eature-**OR**iented **C**omponent **E**ngineering) based on the requirements derived from our exploratory case study. FORCE is based on multiple modeling spaces and supports the hierarchical decomposition and modularization of features models. It provides different kinds of relations and dependencies, as well as feature-to-code mappings. The approach aims at supporting a feature-oriented development process by relating problem-level and implementation-level features. It exploits hierarchies of components with feature models, dependencies between models and feature-to-code mappings. The feature-to-code mappings in the solution space model connect feature model elements to source code elements and define how optional and alternative features are implemented in the program.

**Fig. 5:**
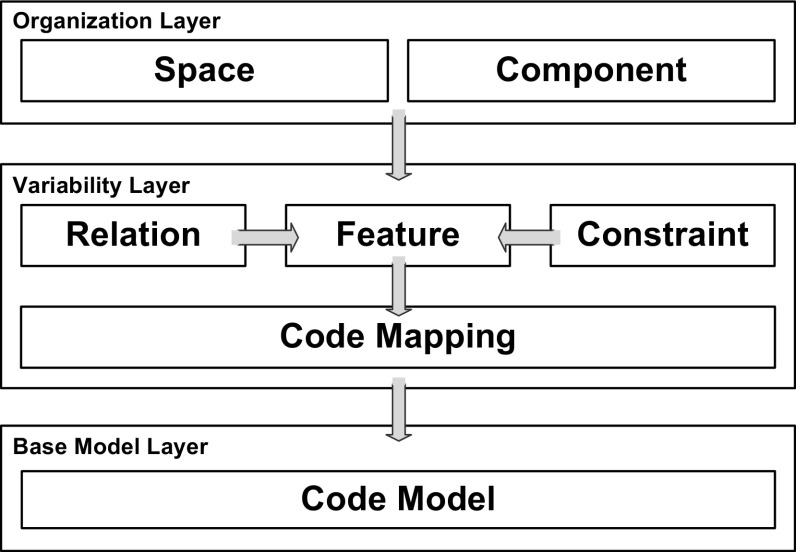
FORCE modeling language architecture

**Fig. 6:**
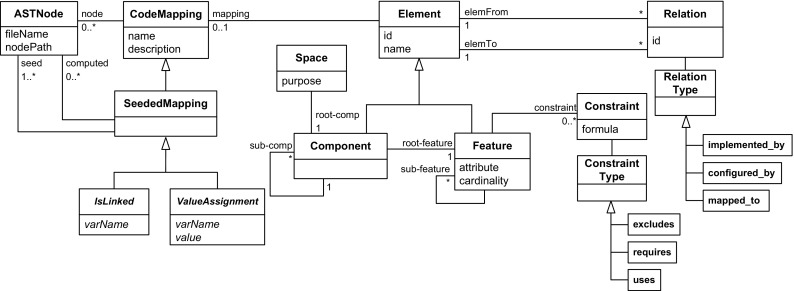
Meta-model comprising core language elements of the FORCE modeling approach. Seeded code mapping types IsLinked and ValueAssignment are introduced in Sect. 7

Figure 5 shows the key language concepts and conceptual layers of FORCE. The *organization layer* covers the language concepts *space* and *component* for structuring feature models both horizontally and vertically. The *variability layer* provides support for modeling *features* defining commonalities and variability. *Relations* and *constraints* support modeling various dependencies within and between models. Further, various types of *code mappings* are introduced for establishing traceability links between the models at the variability layer and the underlying code base. The *base model layer* provides a *code model* of the system. This model can take different forms to represent the system implementation. In our case, it comprises an abstract syntax tree (AST) [29] as well as derived analysis structures. For instance, we use a system dependence graph (SDG) [40] to represent system-wide control and data dependencies in a program [3].

Figure 6 shows the core language elements of the FORCE approach, which we will discuss following the three layers.

### Organization layer

The language elements of this layer allow distinguishing feature models for different purpose. Specifically, FORCE supports multiple modeling *spaces*—each for a distinct purpose—to define the features of a system from different stakeholder perspectives, as suggested by Czarnecki et al. [17]. As our exploratory case study showed, the problem space, configuration space, and solution space provide a foundation for defining the views of product management, product configuration, and software development. However, the set of modeling spaces may be extended or adapted if needed.

*Components* in FORCE adopt the idea of configurable units proposed in the CVL [16]. Components group variability specifications and provide links to related implementations, e.g., software modules. Each component thus comprises a feature model with a root feature as its entry point. For instance, the problem space feature MoldCavityPressureSensor belongs to component Clamp. Our approach further allows structuring components hierarchically to support variability modeling at different levels in each modeling space. Specifically, each modeling space owns a single root component, which may contain several sub-components decomposing a system (cf. Fig. 6). For instance, the mentioned component Clamp belongs to super-component MachineFunctions. Note that such component hierarchies may exist for all modeling spaces and that components or sub-components must not correspond to specific software components (i.e., some components can be used to logically group sub-components). In contrast to the exploratory study (see Sect. 4), we no longer support *collections*, which added unnecessary complexity. Instead, we use *components* as the only mechanism for decomposition.

### Variability layer

The feature models allow defining the product managers’, software architects’, and developers’ understanding of the capabilities and variability of the components. We rely on cardinality-based feature models [20], where features can be arranged into feature groups. Connections between a feature and its sub-features are distinguished as *and*, *or*, and *alternative* groups [5]. The children of *and*-groups can be either mandatory or optional. A feature is either abstract, if not mapped to implementation artifacts, or concrete otherwise [70]. We also use the crosstree constraints requires and excludes already proposed by Kang et al. [41]. Features can be further related via type uses, a special requires relation which describes a code dependency between two feature implementations. This relation type is, for example, useful for expressing that a feature uses library code features. More complex relationships in the form of generic propositional formulas have been proposed in the literature [5] and FORCE also supports crosstree constraints specified in this way.

**Fig. 7:**
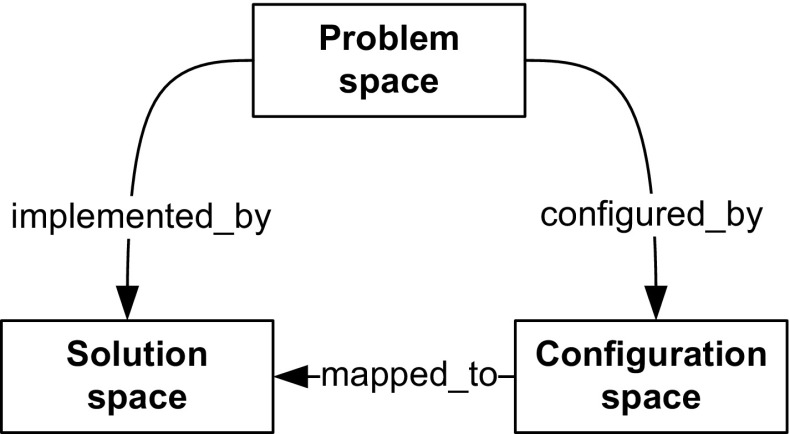
Relations between modeling spaces problem space, configuration space, and solution space

Although FORCE supports building distinct feature models for different spaces representing a system from different perspectives, these feature models are not completely independent. For dealing with such interspace dependencies, FORCE provides the concept of *relations*. Figure 7 shows different relation types we introduced to model relations between problem space, configuration space, and solution space. These relation types are based on the literature. For instance, Heidenreich et al. [36] map features to models describing their implementation. Lee et al. [46] also define implementation dependencies between features. In FORCE, relations of type implemented_by express dependencies between problem space and solution space features, that is, a problem space feature can be implemented by one or more solution space features. For instance, the problem space feature MoldCavityPressureSensor is implemented_by the solution space user interface mask InjectionMask1. To model dependencies between configuration space and solution space features, we use relations of type mapped_to. Dhungana et al. [23] use inclusion conditions to link decisions with assets in DOPLER models. That means, a configuration option (decision in DOPLER) is mapped to an implementation feature (asset in DOPLER) realizing the option. For example, the configuration space option CavityPressureSensor of KePlast ’s configurator is mapped_to the solution space variable hw_CavityPressure. Finally, for documenting dependencies between problem space and configuration space features, we use the relation type configured_by, i.e., for an optional problem space feature there can be a configuration space feature allowing its inclusion. For instance, the problem space feature MoldCavityPressureSensor documented in a product map is configured_by the configuration space option CavityPressureSensor. This relation type is inspired by DOPLER’s visibility conditions controlling which decisions are visible to the application engineer during derivation.

### Base model layer

The code model takes the form of an AST, which represents a program in an object model. *Code mappings* are used to establish traceability from a feature or component to one or more code elements represented as *AST nodes* (cf. Fig. 6). Feature-to-code mappings resemble CVL’s variation points to represent specifications of variability in the software and to establish traceability to related code elements. That means, code mappings are used to link optional and alternative features to code elements implementing the variability, e.g., system variables used to activate code. Code mappings can either be defined manually, e.g., for mandatory features, or semiautomatically via *seeded mappings* [43]. A seed usually is a single code element based on which the mapped elements can be computed, e.g., by analyzing an SDG [3]. For instance, the solution space feature MoldCavityPressureSensor holds a seeded code mapping relating it to the code element Mold1.ai_CavityPressure1. Seeded mappings can be extended to support domain-specific variability mechanisms as we will show in Sect. 7.

## FORCE tool environment

**Fig. 8:**
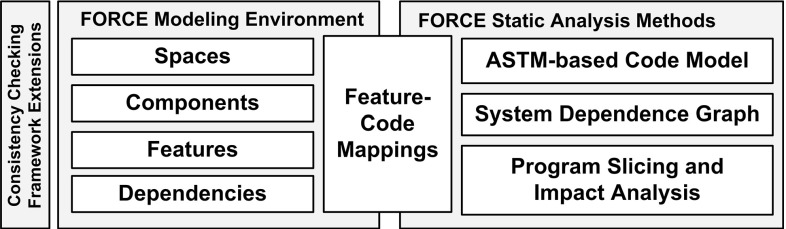
Architecture of the FORCE tool environment

We developed an Eclipse-based tool environment to support the FORCE modeling approach. The overall architecture of the tool environment is shown in Fig. 8. The FORCE modeling environment supports the FORCE modeling language concepts, the FORCE static analysis methods support an AST-based code model and various static analysis methods, e.g., program slicing.

**Fig. 9:**
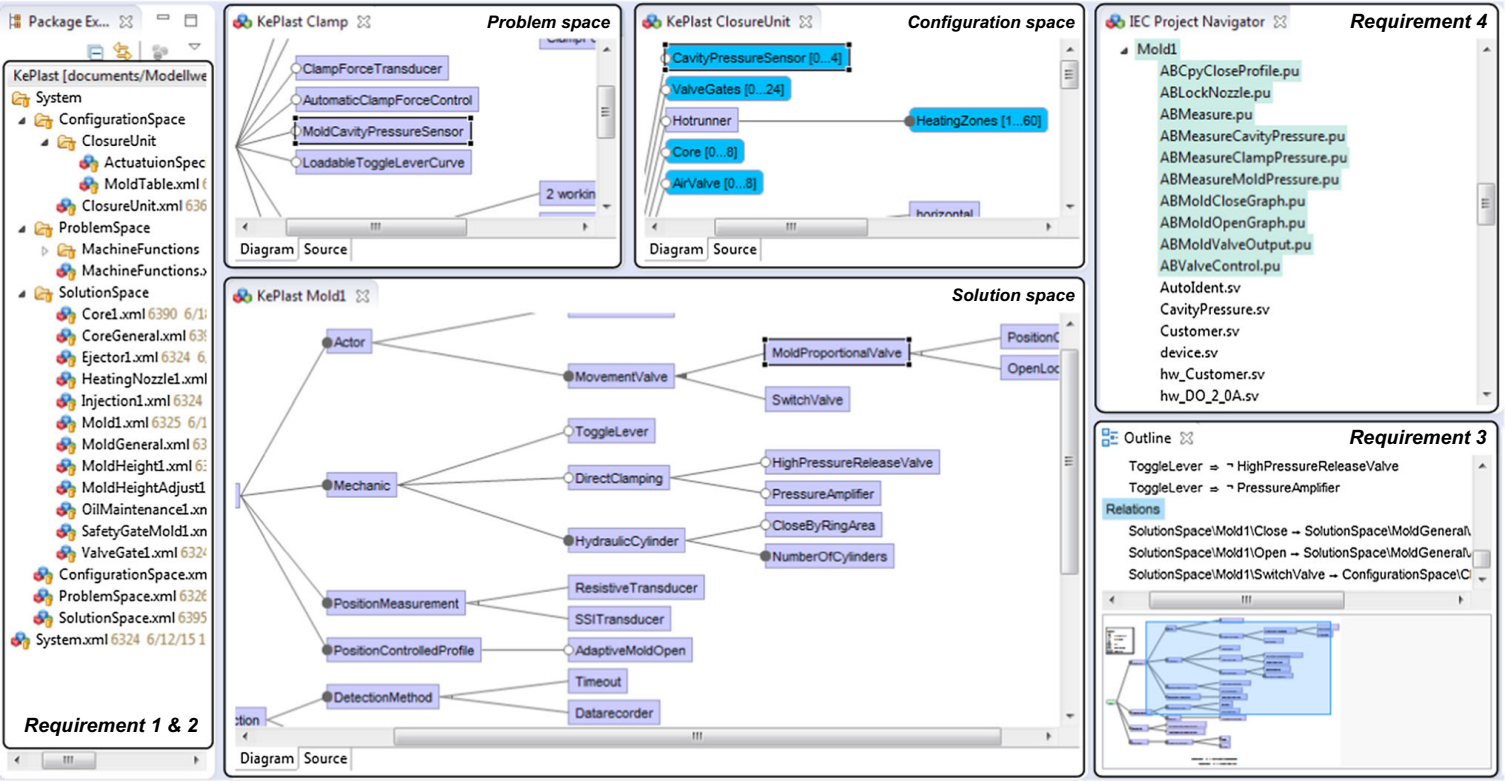
FORCE tool environment showing KePlast feature models

The *modeling environment* has been implemented as an extension of the FeatureIDE [70], a feature-oriented development environment, which is available as an open-source system and easily extensible. The FeatureIDE environment has been extended in various aspects to support the FORCE language: The workspace of the FeatureIDE tool suite can only manage an single feature model. We developed extensions to manage multiple feature models at different levels as well as dependencies between features and components. These dependencies include constraints spanning across feature models and relations types implemented_by, configured_by, mapped_to, as well as uses. The extension points provided by FeatureIDE’s core components did not allow adding new types of modeling elements. Thus, our extensions were mainly done by exploiting inheritance, i.e., the existing model representation of the feature tree was extended to handle the new element types. The existing diagram editor was adapted to display and handle the diagram representations of new element types. We integrated and extended a *consistency checking framework* [61, 71] for determining the consistency of FORCE models. The framework tracks fine-grained change operations to the modeling workspace and triggers consistency checks covering relations and constraints. Engineers are informed instantly and receive feedback about detected constraint violations. We tailored the framework by implementing support for the artifacts needed in our context. Specifically, we instrumented the identified artifacts (feature models and their elements) by developing specific artifact facades for each artifact type. These extensions enable the framework to handle and assign artifacts and contained elements to constraints. We further extended an existing change notifier to inform the consistency checking framework about change operations. Based on the identified artifact dependencies (relations and constraints), we finally developed example consistency constraints. Only affected constraint instances are evaluated by the framework, thus enabling incremental checking.

The *static analysis environment* uses an AST representation of the source code resembling the base model layer of the FORCE language (cf. Sect. 5). The AST model is implemented based on the *Abstract Syntax Tree Meta-Model* (ASTM) [56] standard from the *Object Management Group* and its Modisco implementation [13]. We specialized ASTM for representing the proprietary IEC 61131-3 language dialect used by Keba [33]. Further, the tool environment provides an system dependency graph (SDG) representing all the control and data dependencies in a program, which is build based on the Soot [45] static analysis system. The SDG forms the basis for program slicing and impact analysis, which is needed for computing code-level feature slices [2, 3].

The FORCE tool prototype, depicted in Fig. 9, shows feature models of KePlast. Specifically, it shows feature models for different purposes (requirement 1), at different levels of abstraction (requirement 2), and with different types of dependencies (requirement 3). The Eclipse Package Explorer presents the entry points for the three modeling spaces. For instance, the component Clamp describes problem space features, the component ClosureUnit comprises configuration space features, and the component Mold1 documents solution space features. The FORCE tool environment highlights related code elements (requirement 4) and lists all dependencies (i.e., constraints and relations) available for a selected feature.

## Multiple case study: applying FORCE to PPU and Mold1

We conducted a second case study to validate the capabilities of FORCE. Specifically, we emphasized depth over breadth to cover feature-to-code mappings and consistency rules not covered in our exploratory study. The *objective* of this second case study was to demonstrate the expressiveness and extensibility of the FORCE modeling approach by applying it to different contexts. Specifically, we conducted a multiple case study and applied the FORCE modeling approach to two *cases*: The PPU is a well-known example of a manufacturing system from the literature [73]. The PPU supports material handling, transporting, and sorting of different workpieces. Diverse scenarios describe the PPU’s structure and behavior and its evolution over time [73]. There further exists an implementation covering parts of the PPU comprising about 500 lines of IEC 61131-3 code. The second case is the software component Mold1 from the KePlast product line. Mold1 is responsible for operating an injection molding machine producing plastic parts. The component comprises 15,906 lines of IEC 61131-3 code.

Regarding *RQ3—How suitable is the* FORCE *approach to support multi-purpose, multi-level feature modeling?—*we developed detailed feature models for both systems and exploited FORCE ’s vertical decomposition support to establish traceability links between higher-level problem space and configuration space features with lower-level solution space features. Emphasizing depth over breadth, we modeled dependencies between spaces and used feature-to-code mappings to reveal actual feature implementations. We provide detailed descriptions of problem space, configuration space, and solution space feature models and their characteristics. Regarding *RQ4—How do the extensibility mechanisms of* FORCE *support application-specific feature-to-code mappings and consistency checking of models?—*we analyzed the variability implementation techniques used in PPU and Mold1. We then developed extensions to support the mapping types *IsLinked* and *ValueAssignment*, which use seed variables as a starting point for computing feature implementations using static code analysis techniques. We further developed consistency rules for these case study systems.

### Data collection methods and sources

We obtained qualitative data about the PPU and KePlast systems through archival analysis and workshops.

For the PPU, we studied a technical report by Vogel-Heuser et al. [73], which presents different evolution scenarios of the PPU system, the feature models developed by Bürdek et al. [14], as well as the PPU source code provided by a developer who implemented parts of the PPU. For Mold1, with guidance from Keba engineers (including the authors), we studied the source code of the KePlast system, the custom-developed configuration tool including its implementation [51] and the key KePlast product map. We also collected data in workshops with senior developers, software architects, lead developers, and senior managers from Keba. Specifically, one of the academic authors developed a detailed solution space feature model of the software component Mold1 together with one of the authors from Keba, who has in-depth knowledge of this component. We also collected information on the variability implementation techniques used in Mold1. We further discussed usage scenarios of uses relations with our industry partner. Overall, this allowed us to understand and extract the variability and decomposition mechanisms used in product management, product configuration, and software development.

### Case study phases

We performed a detailed analysis of PPU and Mold1 using the following five steps to complement the coarse-grained analysis of our exploratory study.

*Feature modeling for the three spaces.* We investigated existing artifacts, tools, and source code of PPU and Mold1 to gain insights for each modeling space. For the PPU problem space, we selected the feature model (Version 3) by Bürdek et al. [14]. The solution space model was created by the developer of the PPU control program, based on analyzing the mandatory and variable code parts. The configuration space model was created by collecting and arranging the variabilities of the two other spaces, based on the idea of decision modeling and configuration support [60].

For the problem space of Mold1, we inspected the groupings in the KePlast product map spreadsheet to understand the horizontal and vertical decomposition. The resulting problem space model focuses on the *clamp* part of an injection molding machine, including the platens that provide the force necessary to hold the mold closed during injection of the molten resin and to open the mold for ejecting the molded part. Together with one of the authors from Keba, we created a detailed solution space feature model representing the IEC 61131-3 code of Mold1. To cover the configuration space of Mold1, we focused on the system part *closure unit*, which opens and closes the mold along with ejecting the molded parts. Based on an earlier analysis of the configuration options encoded in Keba ’s custom-developed configuration tool [51], we created a configuration space feature model, which was then refined together with a domain expert.

*Analysis of the variability mechanisms.* The development of the solution space feature model was accompanied by revealing the mechanisms used to implement variability in the code. Specifically, we investigated how variability was implemented for features in the PPU and Mold1 feature models. In case of the PPU system, the feature-to-code mappings were defined manually. For the Mold1 system, we studied different variability implementation mechanisms. For instance, the function IS_LINKED(sv_var:STRING)—testing the presence of system variables in the program—is a heavily used variability mechanism in KePlast as system variables provide links to hardware endpoints.

*Development of tool extensions for the variability mechanisms.* We refined the FORCE tool environment to allow for case-specific variability mechanisms, thus supporting developers to define feature-to-code mappings. For the PPU, we provided manual mapping points allowing the developer to mark optional features in the code. For the Mold1, we provided support for the variability mechanisms *IsLinked* and *ValueAssignment*. We then refined the Mold1 solution space feature model using the seeded code mappings.

*Defining system-specific types of relations and consistency rules.* We further extended our tool to support the additional relation type uses for modeling dependencies between code elements, e.g., that a code part is dependent on library code. We modeled uses relations in the context of PPU and also to define dependencies between Mold1 and its library components. We further defined a set of rules for checking the consistency of relations in our model and implemented selected rules for the two cases in FORCE.

*Review of the feature models including code mappings and relations.* For the PPU system, two authors reviewed the solution space model created by the developer and cross-checked it with the problem space model created by other researchers [14]. The configuration space model was created by one author based on the two other spaces and cross-checked by another author. For the Mold1 system, the authors from Keba reviewed selected parts of configuration space, problem space, and solution space feature models, mappings to code, and dependencies between features to check for completeness, correctness, and consistency.

### RQ3 results

Regarding *RQ3—How suitable is the* FORCE *approach to support multi-purpose, multi-level feature modeling?—*we provide detailed descriptions of the configuration space, problem space and solution space models created for PPU and Mold1 as well as metrics of those models.

**Table 3:** Overview of PPU and Mold1 feature model’s characteristics

Characteristic	PPU	Mold1
Features	29	92
Mandatory	10	23
Optional	9	42
Alternative	10	27
Configuration space	5	44
Problem space	10	14
Solution space	14	34
Components	3	2
Relations	11	15
Constraints	8	12

The PPU problem space model was taken from [14], the PPU solution space model represents the IEC 61131-3 source code related to PPU, and the configuration space model was created based on the two other spaces. The Mold1 problem space model focuses on an injection molding machine’s *clamp*. The Mold1 configuration space models refer to an injection molding machine’s *closure unit*. The solution space model represents the IEC 61131-3 source code related to Mold1

Table 3 shows metrics of models for PPU and Mold1 in terms of number of components, features, relations, and constraints. For PPU, the problem space model was taken from [14] and comprises ten features not including the root feature. The solution space model represents the IEC 61131-3 source code and includes two sub-components, 14 features, ten feature-to-code mappings, and eight uses constraints reflecting the dependencies in components Stack and Crane. The configuration space model was created based on the two other spaces to reflect the possible configuration choices. It comprises three optional and two alternative features.

**Fig. 10:**
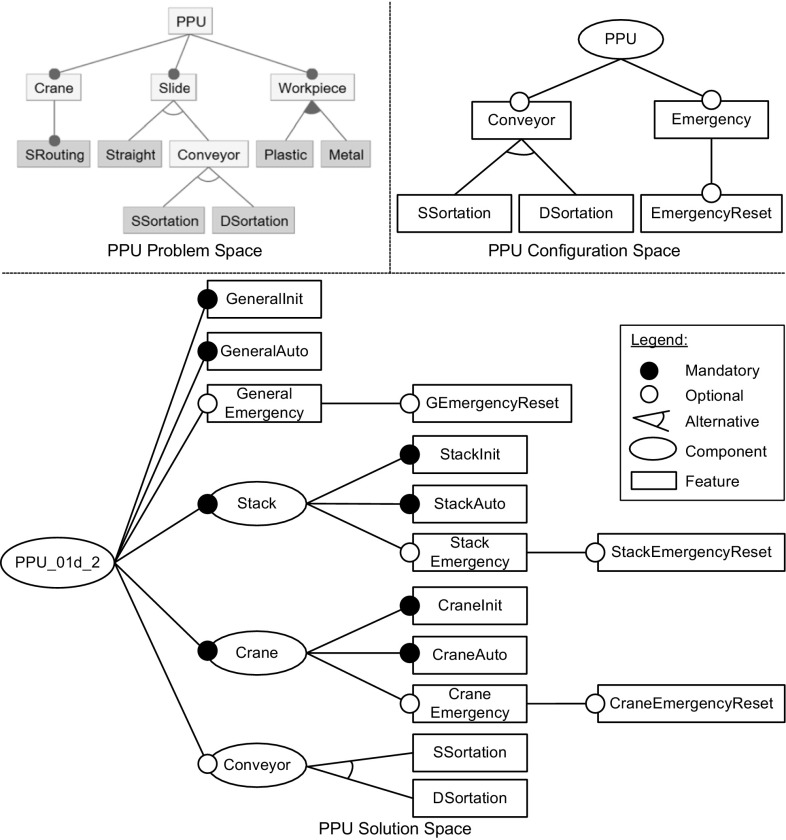
Problem space feature model of PPU (taken from [14]), configuration space feature model of PPU and solution space feature model of PPU snapshot 01d_2

Figure 10 shows the problem space, configuration space and solution space feature models of PPU. The problem space model represents the system from the perspective of customers. The system’s main elements are the crane and the output device for transportation of workpieces. The main choices cover the used output device which can either be purely mechanical or a conveyor. Conveyors allow sorting workpieces, using the strategies SSortation or DSortation. Moreover, plastic or metal workpieces may be handled. The solution space model reflects the structure and capabilities at code level. Note that the Conveyor component is optional, i.e., no software component is needed in the case the pure mechanical output device is used. If the Conveyor component is used, two alternative features represent the sorting strategies. The main optional features in the software are if emergency and emergency reset are supported. Elements regarding those options can be found in the main component as well as in sub-components Stack and Crane. The solution space model also models mandatory features for initialization and automatic mode. Mandatory features are important to represent how features are implemented using feature-to-code mappings. Note that the material of the workpieces (plastic and metal) is not reflected in the solution space model as the control software always supports both types. Finally, the configuration space model contains the choices in the system on using a conveyor, selecting a sorting strategy, and defining the preferred emergency support.

The PPU models comprise 11 interspace relations. For instance, the configuration space feature Emergency is mapped_to the solution space features GeneralEmergency, StackEmergency and CraneEmergency. Further, the problem space features SSortation and DSortation are configured_by the configuration space features SSortation and DSortation and implemented_by the solution space features SSortation and DSortation.

**Fig. 11:**
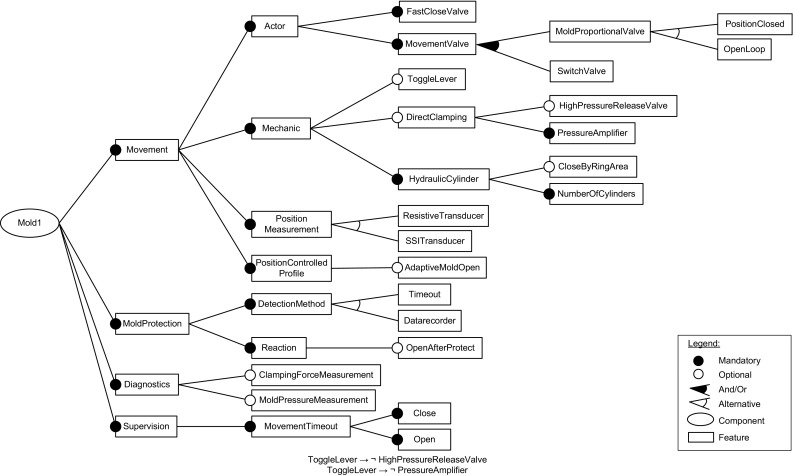
Solution space feature model of subsystem Mold1

For Mold1, the resulting problem space feature model comprises 14 features related to the clamp of an injection molding machine. The created configuration space feature models reflect the closure unit of an injection molding machine and include two sub-components and 44 features and 5 features with cardinality. The resulting solution space feature model contains 34 features, 19 mandatory, 6 alternative, and 9 optional.

Figure 11 shows the solution space feature model of Mold1. The top features (shown beneath the Mold1 component) represent group features used for organizing the model into meaningful parts. Thus, the feature group Movement comprises features related to opening and closing the mold along with ejecting the parts and the mechanic type of mold movement. This feature group has a rich set of sub-features further grouping features and defining different alternatives and options. So Actor relates to different valve types used in the hydraulic system (e.g., MoldProportialValve or SwitchValve). Within the Mechanic group, different alternative and optional equipment is modeled. For example, two optional machine types are ToggleLever and DirectClamping, where toggle lever systems are actuated by hydraulic cylinders utilizing mechanical linkages to generate higher forces than a direct connection from a hydraulic cylinder of the same size. Furthermore, there are alternatives for measuring the mold position ( ResistiveTransducer and SSITransducer) and a feature for controlling the mold position based on a given profile ( PositionControlledProfile with option AdaptiveMoldOpen). The feature group MoldProtection comprises features for supervising the closing of the mold, especially until the two platens are touching. Its functions are used for detecting objects between the two platens, which could damage the mold. As can be seen, there are two alternative detection methods, time-based (without sensor) and datarecorder (using a dedicated mold protection sensor) modeled as alternative features TimeOut and Datarecorder. The feature Reaction modeling the response in case of problems has a sub-feature OpenAfterProtect which is optional. The feature group Diagnostics defines two sub-features, evaluating clamping force and mold pressure values, respectively. As shown by the model diagram, both those features are optional. Finally, the feature group Supervision contains features for detecting problems, e.g., unwanted movement when the mold should be stopped, or no movement when the mold should move. Those features are all mandatory and therefore included in any product. Furthermore, we created 14 feature-to-code mappings for solution space features (cf. Table 4).

**Table 4:** Code mappings created for the Mold1 feature model. The parameters define variable names, variable values, and slice directions used

Feature	Code mapping type	Parameter
FastCloseValve	IsLinked	Mold1.do_FastClose
MoldProportionalValve	IsLinked	Mold1.ao_Valve
PositionClosed	Manual	backward slice hw_Mold1.sv, Pressure.sv, MoldControl.sv
OpenLoop	Manual	backward slice MoldValve.sv, hw_Mold1.sv, Valve.sv
ToggleLever	Manual	forward slice Mold1.sv_Options AND cSubOptionMoldDirectLock
DirectClamping	Manual	forward slice NOT(Mold1.sv_Options AND cSubOptionMoldDirectLock)
HighPressureReleaseValve	ValueAssignment, IsLinked	Mold1.do_OpenHighPre = true, Mold1.do_OpenHighPre
PressureAmplifier	ValueAssignment, IsLinked	Mold1.do_PressureAmplifier = true, Mold1.do_PressureAmplifier
CloseByRingArea	ValueAssignment	Mold1.sv_CylinderData.bUseSmallSize = true
PositionControlledProfile	Manual	backward slice Mold1.sv_MoldBwdProfVis, Mold1.sv_MoldFwdProfVis
TimeOut	Manual	backward slice Mold1.sv_dMoldProtectTimeSet, Mold1.sv_dMoldProtectTimeAct
OpenAfterProtect	ValueAssignment	Mold1.sv_bOpenAfterProtect = true
ClampingForceMeasurement	IsLinked	Mold1.ai_ClampPress
MoldPressureMeasurement	IsLinked	Mold1.ai_CavityPressure1

The Mold1 models comprise 12 constraints: Four uses constraints have been modeled to express code dependencies in the solution space model. For example, the features Open and Close use library features to operate mold movements. Two constraints listed in Fig. 11 are related to ToggleLever systems. The features HighPressureReleaseValve for defining the pressure release end position during mold open via the user interface, and PressureAmplifier including the force threshold for activating the pressure amplifier are only available for systems using direct clamping. And finally, six constraints related to the configuration space feature models of Mold1 were extracted by one of the authors by inspecting Keba ’s configuration tool. These constraints document that specific production modes require different sensors and valves.

The Mold1 models then comprise 15 interspace dependencies of type configured_by and implemented_by, i.e., documenting that the problem space feature MoldCavityPressureSensor is configured by configuration space feature CavityPressureSensor and implemented by the solution space feature MoldPressureMeasurement. Further relations of type mapped_to link the configuration space feature CavityPressureSensor to specific components of the KePlast visualization system.

### RQ4 results

Regarding *RQ4—How do the extensibility mechanisms of* FORCE *support application-specific feature-to-code mappings and consistency checking of models?*—we provide details on the code mappings we created for Mold1 features and the extensions of the mapping types for handling application-specific variability mechanisms. For PPU, all mappings are manual mappings and a developer created ten feature-to-code mappings of type Manual for PPU. Thus, those are not further presented. We further provide examples of constraint rules using FORCE ’s dependencies. We present how such rules can be stated as consistency constraints, and finally, we describe how constraint violations are presented to engineers.

Developers use a wide range of variability mechanisms as shown in a recent paper [75]. To cope with the specific variability mechanisms used in KePlast, we developed two special mapping types. *IsLinked* is a frequently used variability mechanism. Program variables usually represent the endpoints to hardware equipment, e.g., a variable is used to provide the values of a sensor. For optional hardware equipment, the variable declaration is used as variation point in the software. Further, the program will contain conditional statements testing whether the variable declaration is present (using built-in function IS_LINKED) and only then conditionally execute the code responsible for handling that optional equipment. For modeling this type of variability mechanism, we have defined a special seeded mapping type *IsLinked,* where a program variable serves as the seed. Then, static analysis collects all code parts which are conditionally executed on this presence condition and recognizes them as belonging to the mapped code elements (cf. [3]).

**Table 5:** Examples of constraint rules (CR) in FORCE

Id	Constraint	Informal Description
CR1	Relations configured_by and mapped_to imply implemented_by	If a problem space feature is configured_by a configuration space feature, which is also mapped_to a solution space feature, then the problem space feature must be related to this solution space feature via implemented_by (see also Algorithm 1)
CR2	Relations mapped_to and implemented_by imply configured_by	If a configuration space feature is mapped_to a solution space feature and a problem space feature is implemented_by this solution space feature, then the problem space feature must also be configured_by the configuration space feature
CR3	Completeness of solution space features	A solution space feature is only complete if it defines a code mapping
CR4	Completeness of configuration space features	A configuration space feature is only complete if it is related to at least one solution space feature via mapped_to
CR5	Consistency of optional features with code	Optional solution space feature are only consistent with the code if they are mapped to code which is dependent on a configuration, e.g., conditionally executed
CR6	Consistency of alternative features with code	Alternative solution space features are only consistent with the code if they are mapped to code which is dependent on a configuration, e.g., conditionally executed
CR7	Interspace constraints contradictions	If configuration space or problem space features exclude each other, the referenced solution space features may not require or use each other. Analogously, if configuration space or problem space features require each other, the referenced solution space features may not exclude each other
CR8	Usage of the uses relation	A uses relation can only be defined between solution space features and may not be used between problem space and configuration space features
CR9	Usage of mandatory features in configuration space	Leaf features in the configuration space must not be mandatory

A further conditional execution variability mechanism used in the KePlast software system is to exploit configuration variables. Similarly to the *IsLinked* variability mechanism, conditional statements will test those configuration variables for specific values and thus enable or disable the code implementing the respective option. The mapping type *ValueAssignment* allows specifying this kind of variability mechanism. As for the *IsLinked* type, static code analysis can be used to find all the conditional statements, by testing the variable for the specific value and recognizing the code elements in the respective branch as mapped elements. Engineers create code mappings semiautomatically as they can finally inspect and possibly adapt the computed mappings by adding or removing code elements. Besides these two seeded mapping types, it is possible to define mappings manually by selecting specific code parts, e.g., for mandatory features.

Table 4 summarizes the mappings we have created in the Mold1 feature model and the types of mappings used. For instance, the Mold1 feature model contains a code mapping of type IsLinked for feature MoldProportionalValve. This feature defines the valve used for mold positioning. If PositionClosed mold movement is selected, mold positioning is controlled by a servo valve requiring an additional pressure sensor. If OpenLoop mold movement is selected, a proportional valve is used for mold positioning. The qualified variable name used for the code mapping is Mold1.ao_Valve. The code mapping related to feature CloseByRingArea is an example of type ValueAssignment. The feature CloseByRingArea calculates the effective force of the hydraulic cylinder depending on the orientation of the cylinder, the area of the piston and the rod. Thus, the option is enabled by if-condition Mold1.sv_CylinderData. bUseSmallSize = true. The code mapping for the feature ToggleLever is an example of a mapping of type Manual, i.e., a developer has to define the code belonging to that feature. However, she is supported by static analysis methods. First, she maps the variable mbDirectLock to the feature. Then, she can compute a forward slice to determine the code dependent on this variable. Finally, she can select the statements she wants to definitely map from the slice.

**Fig. 12:**
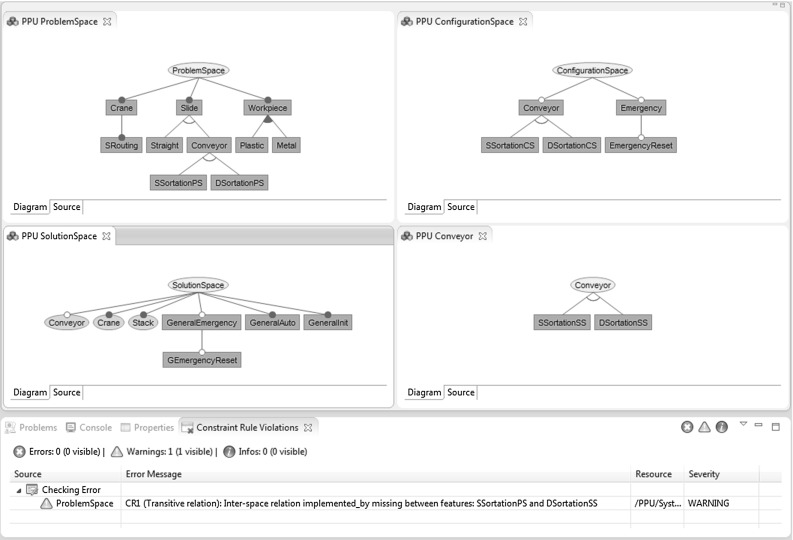
Tool prototype showing PPU’s problem space, configuration space, and solution space feature models. Features SSortationPS, SSortationCS, and SSortationSS are related via configured_by and mapped_to relations. However, the implemented_by relation between features SSortationPS and SSortationSS is missing (as shown in the view Consistency Rule Violations) which is evaluated by the constraint implementation outlined by Algorithm 1

Consistency checking within and between features models requires the definition of application-specific constraints. FORCE can easily be extended with constraint rules to check dependencies in models. We present examples of such consistency constraints and describe how constraint violations can be presented to engineers. Table 5 lists examples of constraint rules (CR) relevant in FORCE. For instance, rules CR1 and CR2 observe the interaction of interspace relations in their entirety. These rules inform the engineer about missing relations between spaces. The constraint implementation outlined by Algorithm 1 ensures that rule CR1 holds at the level of features. More specifically, the consistency checking framework evaluates the constraint when a problem space feature is changed (e.g., when a new relation is added). Figure 12 shows the evaluation result for PPU’s feature models: The features SSortationPS, SSortationCS, and SSortationSS are related via configured_by and mapped_to relations. However, the implemented_by relation between features SSortationPS and SSortationSS is missing (as shown in the view Consistency Rule Violations). However, despite this inconsistency, the engineer is not hindered to continue her work and, e.g., may fix the inconsistency by propagating the modification to all related clones. In this regard, our approach follows Balzer’s idea of tolerating inconsistencies [4].
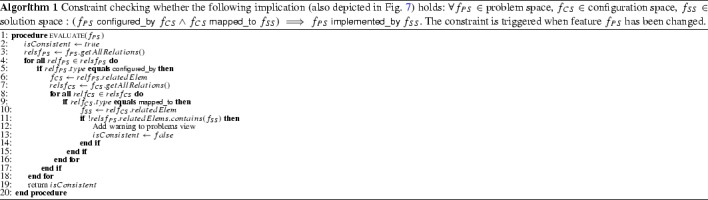


Further, rules CR3 and CR4 deal with completeness issues. Rule CR3 ensures that each feature in the solution space is mapped to code elements. Analogously, rule CR4 checks that configuration space features are mapped to solution space features. Note that no rule regarding completeness of problem space feature relations exists. The investigated cases show that some problem space features are used for communication with customers only and thus do not have corresponding relations to configuration space and solution space features.

The rules CR5 and CR6 take care that the variability as modeled in the solution space is consistent with the implementation and rule CR7 checks that constraints in solution space and constraints in problem space and configuration space models do not contradict. Finally, rule CR8 restricts the usage of the uses relations to solution space space features and rule CR9 ensures that leaf features in the configuration space are not mandatory.

The list of constraint rules is not exhaustive; however, the consistency checking framework allows to be extended regarding new constraints. FORCE currently supports rules CR1, CR3, CR4, CR8, and CR9. Thus, engineers are warned if they, for instance, model a solution space feature without specifying a feature-to-code mapping, or if a configuration space feature is not mapped_to to a solution space feature, or if uses relations include problem space and configuration space features, or if configuration space models contain mandatory leaf features.

### Summary

Regarding RQ3, we have developed feature models for PPU’s and Mold1 ’s different modeling spaces, this time emphasizing depth over breadth and feature-to-code mappings. We have shown that FORCE allows building feature models in problem space, configuration space and solution space for PPU and Mold1. In particular, we have built and depicted PPU’s feature models and a detailed feature model of Mold1 ’s solution space. FORCE allowed modeling the dependencies between features of different components we encountered during the case study. We thus conclude that FORCE is sufficiently expressive to support multi-purpose, multi-level feature modeling. We also learned that there is currently a lack of explicit knowledge about feature dependencies, so we see the models only as a first step toward a richer set of models documenting interspace dependencies, which may require the introduction of additional types of relations. We thus conclude that we successfully demonstrated the application of the FORCE approach to the PPU system and Mold1 component of KePlast.

With respect to RQ4, we demonstrated that FORCE is sufficiently extensible to deal with application-specific variability mechanisms and consistency checks. Specifically, we have shown feature-to-code mappings specific to the variability implementation mechanisms used in PPU and Mold1, showing that our approach can be customized to different needs. We further showed how consistency checks can be added to FORCE, which exploit the relations and constraints defined within and between different feature models in FORCE. We further showed how constraint violations can be presented to engineers.

## Discussion

We summarize observations and lessons learned we made when modeling and validating the feature models in the two case studies. We also discuss threats to validity.

### Being specific about the purpose and level of features facilitates modeling

Our modeling approach is based on classifying features according to their purpose to better understand their role in the system. The approach further allows defining features at different levels of granularity. The feature models created in our exploratory study comprise more than 100 structural elements in all modeling spaces, thus confirming the need and usefulness of modularizing feature models in such a divide-and-conquer manner. The use of different modeling spaces supports involvement of modelers with different background, as they can focus on their area of expertise, i.e., product management, architecture, or product configuration aspects. Our results from the second case study further show that detailed domain expertise is required for defining the feature models, regardless of the modeling space. Typically, models could only be created after detailed inspections of other artifacts such as product maps, models, or source code.

### Feature models help to limit variability

Feature models have originally been proposed to elicit and represent variability *and* commonalities of systems’ capabilities [42]. Feature models show explicitly what is *not variable* and which product variants are *not possible*. Keba uses a wide range of variability mechanisms. In FORCE, the feature models define a variability interface to components, which helps to control the otherwise unlimited flexibility, thus improving guidance for developers.

### Focusing on the dependencies between feature models helps developing a system-wide perspective

The feature models cannot be defined in isolation, and understanding their dependencies is fundamental in a feature-oriented development process. However, revealing and understanding dependencies between features from different models turned out to be extremely challenging as can be seen by the rather low numbers of dependencies in both case studies. Our observation is supported by Berger et al. [9], who found that modelers in industry focused on building the parent–child relationship between features, while trying to avoid crosstree constraints. It has been pointed out that modeling dependencies would be very helpful, e.g., to reveal the implementation of high-level features in the code base, or check consistency during product derivation [54]. However, while providing modeling support for dependencies is easy, revealing actual dependencies between modeling spaces, and people working with features in these spaces, is much harder. Again, involving different roles is useful: Software engineers in charge of a component can define its solution space features, while system architects can define dependencies between different feature models. In this context, FORCE ’s support for ensuring the consistency of model dependencies can help modelers to find missing dependencies, e.g., when receiving notifications about violations of rules CR1 and CR2.

### Supporting modelers by suggesting certain features automatically

Depending on the purpose and level of feature models, we found potential to support modelers with feature suggestions to at least partially automate the creation of models. Problem space models can be created by analyzing product comparison matrices, as, e.g., recently demonstrated by [6]. The product maps of Keba follow a similar structure, and supporting modelers in creating initial feature models via suggestions would be feasible. Our program analysis capabilities [2, 3, 33] also support populating solution space models, including the identification of uses constraints across different components. This will be part of our future work. Similarly, configuration space models can be computed by analyzing variability at solution space level, cf. configuration space model of PPU in Fig. 10. While this can streamline the modeling process, it has been shown that configuration models go beyond simply presenting configuration choices and particularly need to ensure user guidance [60], so full automation is often not possible.

### Threats to validity

As with any empirical research, our results may not generalize beyond the cases we considered. There is a potential bias caused by the systems KeMotion and KePlast and component Mold1 selected for the evaluation, as they are all from the industrial automation domain. However, the systems are from two different areas (i.e., injection molding and robotics). The KePlast component Mold1 was suggested as a typical component by a software architect of Keba. We also try to avoid generalizations and present a detailed analysis of the models we created. Moreover, the PPU system used as the second case represents an example outside the company and is regarded as a standard example representative for the domain.

We only present descriptive model metrics and cannot claim statistical significance. In particular, it can be argued that the number of modeled dependencies is rather low. However, the current lack of explicit knowledge on feature dependencies is an interesting finding of our study. Overall, given that companies typically do not provide access to data about their systems, we believe that our results are valuable to other researchers and practitioners.

Some authors of this paper made significant contributions when creating the feature models, it can thus be argued that the results are solely due to our manipulations. However, the author of Keba creating the models for the initial study adhered to product maps, specification documents, custom-developed configurators, and the code base, mature artifacts created and maintained by diverse domain experts without any influence from our side. The feature model representing the component Mold1 was developed together with an author from Keba having in-depth domain knowledge. The resulting Mold1 feature model thus captures expert knowledge related to injection molding machines and the domain expert decided how to structure and shape the model without any influence from our side. For PPU, we followed the descriptions and models as found in the literature [14, 73], and implementation and models were created by a developer not part of the author team. We further attempted to mitigate this threat by performing an iterative and joint modeling process involving both the academic and industrial authors, to benefit from feedback and validation based on prototypes of the models.

## Related work

Variability modeling is a core activity in software product line engineering (SPLE) [15], and a wide range of variability modeling approaches have been proposed, including feature modeling [41], decision modeling [66], and orthogonal variability management [58]. We discuss existing case studies on variability modeling, research on modularization, multi-product lines and megamodels, approaches for modeling dependencies between modeling spaces and feature-to-code mappings.

### Case studies on feature modeling in practice

Several empirical studies exist on applying feature modeling in practice; however, only few reports exist on variability modeling in large-scale systems. For instance, Berger et al. [10] provide a detailed analysis of features in 128 variability models including detailed metrics about feature types, numbers of features, and feature dependencies. The authors further perform a deep qualitative analysis of 13 models, which also addresses several topics covered by FORCE. For instance, the study showed that modularization is frequently used when aiming at scaling variability modeling. Further, similar to FORCE ’s modeling spaces, the analysis showed a separation of development and configuration views. Lee et al. [47] report detailed modeling experiences related to an elevator control software product line comprising 490 features—157 capability, 22 operating environment, 291 domain technology, and 20 implementation technique features. The feature spaces used by Lee et al., originally proposed by Kang et al. [42], are related to the modeling spaces we used in our approach: Capabilities are addressed by configuration space and problem space features. Domain technologies are reflected by solution space features; however, some problem space features also address domain technologies. The operating environment is related to configuration space features, e.g., specific hardware equipment of injection molding machines or robots. Developers are concerned about specific implementation techniques, which are covered by solution space features. Finally, a recently conducted case study provides an in-depth analysis of 23 features in real-world settings based on interviews investigating the practical use of features in three large companies [8]. The authors use feature facets for describing and comparing features. Some of the facets are related to the issues explored in our paper. For instance, the facet *use* relates to the purpose of feature models, and the *position in hierarchy* is related to the modeling level.

### Modularization and multi-product lines

MultiDeltaJ is an approach to represent delta-oriented multi-product lines covering problem, solution, and configuration space [21]. The programming approach aims at obtaining multi-product lines by fine-grained reuse of delta-oriented product lines. Like FORCE , MultiDeltaJ supports hierarchies and modular structures. Similar to the components in our approach, Kästner et al. propose a variability-aware module system, enabling a divide-and-conquer strategy to software development and breaking with the anti-modular tradition of a global variability model in product line development [44]. Modules are considered as product lines, which can be type checked in isolation; however, variability can crosscut multiple modules. Dhungana et al. [24] present an approach that aims at reducing the maintenance effort of modeling product lines by organizing the modeling space as a set of interrelated model fragments defining the variability of particular parts of the system. Our approach also aims at modularizing feature models, to support the distributed development and modeling of components. Holl et al. [38] support multiple users in performing distributed product derivation of a multi-product line by sharing configuration information. Their approach uses product line bundles (PLiBs) for organizing and deploying product line models and domain-specific tools [72]. Specifically, a PLiB serves as a container and packages variability models together with tool extensions and settings such as organizational policies or expiration dates of models. As stated above, in this work we focus on modeling support. However, the concept of PLiBs could be useful when using FORCE models in a distributed work mode. The CVL [16] is a domain-independent language proposal for specifying and resolving variability. It facilitates the specification and resolution of variability over any instance of a Meta-Object Facility (MOF)-based language, which is termed a base model. Configurable units are an integral part of CVL and are used for grouping associated variation points. FORCE ’s components adopt the idea of CVL’s configurable units. The base variability resolution (BVR) language builds on CVL but provides extensions relevant for industry [34]. For instance, BVR supports references, logical relationships, and groups and discusses further concepts supporting recurring patterns. Such recurring patterns have also been found in Keba ’s systems. For instance, to support several cores in injection molding machines, engineers typically create multiple component instances based on a component template.

### Mega modeling

Bézivin et al. [11, 12] have recognized the need for global model management using megamodels, i.e., composites of interrelated models and meta-models for describing large-scale systems. Megamodels consider models as first-class citizens, and relevant dependencies are, for instance, the conformance relation between a model and its meta-model. The Atlas Mega Model Management approach (AM3) provides practical support for developing megamodels [1]. Similarly, Salay et al. [65] introduce macromodels for managing multiple models at a high level of abstraction expressed in terms of models and their intended relationships. Seibel et al. [67] present dynamic hierarchical megamodels combining traceability and global management. Another topic of interest in multi-modeling is checking model consistency. Denton et al. [22] present the NAOMI platform for managing multiple models developed in different modeling languages. The approach analyzes dependencies to determine the impact of changes on dependent models and to propagate changes. As our results show, components in our approach can be seen as individual models used for defining features of large-scale systems. However, although we manage dependencies between different modeling spaces, thus relating models similar to the approaches above, we focus on relations between individual features in these components rather than dependencies between models.

### Modeling of dependencies

Many approaches emphasize modeling dependencies between different modeling spaces. For instance, FeatureMapper and VML* support modeling the relationship between problem space features and solution space models describing product line details (e.g., requirements models, architecture and design models) [37]. However, these approaches do not take configuration space features into account, which comprise around 30 % of features in both KeMotion and KePlast feature models. The COVAMOF [68] framework models variability in terms of variation points and variability dependencies at different levels of abstraction (i.e., features, architecture, and implementation). COVAMOF uses realization relations for providing a hierarchical organization of variation points. In contrast, our approach supports nesting feature models to build hierarchical models. Furthermore, COVAMOF’s dependencies focus on guiding and restricting the selection of variation points during product derivation. The consistency checking approach used in FORCE currently primarily supports engineers creating feature models of large-scale industrial systems. Dependencies are also important in multi-level feature trees, an add-on to traditional feature models that introduce the notion of reference feature models, which serve as a template and guideline for the referring model [62]. The reference model becomes a means to strategically drive the content of the referring model by allowing or disallowing certain deviations. Locally introduced innovations can be made globally visible in a step-by-step process. Although the FORCE modeling language does not not provide guidelines in form of reference models, we have recently been working on extensions allowing to use feature models in a clone-and-own development process that relies on compliance levels between the original feature model and its clone [59].

Our work on consistency checking also relates to research on checking the consistency of requirements and requirements dependencies. Nuseibeh et al. [55] present an approach based on multiple ViewPoints holding partial requirements specifications. The authors propose a general model for ViewPoint interaction and integration and present the notion of inter-ViewPoint communication in the context of a ViewPoints framework. They also elaborate on inter-ViewPoint relationships as vehicles for consistency checking and inconsistency management. Goknil et al. [32] provide formal definitions of commonly used requirements relation types. These definitions are used for consistency checking of requirements relations and for inferring new relations. Their primary traceability goal is change impact analysis, e.g., determining which model elements are impacted by changed requirements. Zowghi and Offen [76] present a logical framework for modeling and reasoning about the evolution of requirements. They demonstrate how a sufficiently rich meta-level logic can formally capture intuitive aspects of managing changes to requirements models, while maintaining completeness and consistency.

### Feature-to-code mappings

Specifying feature-to-code mappings is an essential task in our modeling approach. Therefore, our FORCE tool environment has built-in support for determining the code of features in a semiautomatic way. Numerous approaches have been proposed to support developers in locating implementations of features in code. First, software traceability techniques have been proposed to recover trace links between requirements, features, and code [25, 27]. For example, Fischer et al. [30] presented an approach to compute feature-to-code mappings by correlating differences in product configurations to differences in source code. However, their approach assumes that product variants do not contain dead code which is not the fact in our case study. Despite successes in this field, trace recovery remains a human-intensive activity. Indeed, researchers have pointed out that it is risky to neglect humans in the traceability loop [35] and studies exist on how humans recover such traces manually [28].

Kästner et al. [43] presented an approach that uses a variability-aware type system to assist developers to detect features in source code. They also use a *seeded* approach together with a variability-aware type system. However, our approach uses program slicing starting at defined seeds to find a possible set of statements. In our context, finding the initial seed means defining a variation point which requires human expertise and domain knowledge. Kästner et al. also leverage domain knowledge for finding seeds and additionally to define and compute the relations between features. Although similar techniques are used, the main difference is that our approach aims to find the implementation of a feature and not to mine features in source code.

Petrenko et al. [57] introduced an approach called *JRipples* that also uses a dependency graph to find program elements starting at certain seeds. A user manually decides, which dependencies still need to be investigated and systematically follows the dependencies. JRipples especially supports several granularities to reduce the effort for the user. Our semiautomatic approach works in a very similar way. A user starts at seeds, and all dependent elements are added by default. The user can then remove or add further elements manually. However, we do not explicitly support different granularity levels because this is implicitly supported since our elements are nodes in the AST. If a higher-level node, e.g., a class, is added, all children are also added implicitly.

## Conclusion and future work

This paper presented an approach and experiences of applying a multi-purpose, multi-level feature modeling approach to two large-scale industrial automation systems. The paper first reported results and experiences of an exploratory case study investigating the characteristics and modularity of the feature models and their dependencies. The findings allowed developing FORCE, a modeling approach that extends an existing feature modeling approach to support models for different purposes and at multiple levels, including traces to the underlying code base and consistency checks. Our feature modeling environment extends the FeatureIDE modeling tool and is integrated with static code analysis and consistency checking frameworks. We then demonstrate the expressiveness and extensibility of our approach by applying it to a well-known example of a manufacturing system for material handling and sorting of different workpieces and an injection molding subsystem of an industrial product line. Overall, our results and experiences show that considering the purpose and level of features is useful, that understanding dependencies between feature models is essential for developing a system-wide perspective, that code-level views and domain dictionaries are important to understand the meaning of features, and that feature models help to limit otherwise boundless variability.

## Footnotes

1 Daniela Rabiser’s previous work was published under the name Daniela Lettner.
